# Correlation Between Kidney Function and Intestinal Biological Characteristics of Adenine and *Folium Sennae*-Induced Diarrhea Model in Mice

**DOI:** 10.5152/tjg.2022.211010

**Published:** 2023-01-01

**Authors:** Xiaoya Li, Jiayuan Zhu, Yi Wu, Zhoujin Tan

**Affiliations:** 1 College of Chinese Medicine, Hunan University of Chinese Medicine, Changsha, China; 2 College of Pharmacy, Hunan University of Chinese Medicine, Changsha, China; 3 College of Medicine, Hunan University of Chinese Medicine, Changsha, China

**Keywords:** Adenine, diarrhea, Folium sennae, intestinal enzyme activities, kidney function, microbial activity

## Abstract

**Background:**

This study investigated the correlation among kidney function, intestinal enzyme activities, and microbial activity of adenine and *Folium sennae*-induced diarrhea model in mice, which provided a basis for clinical treatment of kidney-intestinal correlation.

**Methods:**

We performed different doses of adenine combined with *Folium sennae* intragastric administration to establish the animal model of diarrhea. We assessed thymus and spleen indexes, serum creatinine, urea nitrogen and uric acid contents, intestinal contents and mucosal enzyme activities, and microbial activity.

**Results:**

After modeling, mice presented increased serum creatinine and decreased urea nitrogen. Uric acid showed different changes in the different model groups. The thymus index in the model mice was trending downward, whereas the spleen index was the opposite. Moreover, model mice induced a non-significant increase in xylanase activity of the intestinal contents and mucosa compared to the control performance. Sucrase content of the intestinal contents increased considerably in the model groups but decreased in the intestinal mucosa. Lactase and amylase induced different trends in the different modeling methods. As well, the microbial activity of intestinal contents increased significantly, while that of intestinal mucosa decreased.

**Conclusion:**

Adenine combined with *Folium sennae* successfully replicated diarrhea in mice models. Using 50 mg/ (kg/day) adenine for 14 days in combination with 10 g/(kg/day) *Folium sennae* decoction for 7 days caused kidney function injury in diarrhea mice. In addition, kidney function injury was accompanied by changing in intestinal functional enzyme activity and microbial activity.

**Main Points**

- Adenine combined with *Folium sennae* successfully replicated diarrhea in mice models.
- Using 50 mg/(kg/day) adenine for 14 days in combination with 10 g/(kg/day) *F. sennae* decoction for 7 days caused kidney function injury in diarrhea mice.
- Kidney function injury increased the metabolic capacity of intestinal contents and decreased the metabolic capacity of intestinal mucosa microorganisms.
- Kidney function injury was accompanied by changing in intestinal contents and mucosal functional enzyme activities in mice.

## Introduction

Diarrhea is a common gastrointestinal disease characterized by increased frequency of feces and loose or watery feces.^1^ According to its clinical manifestations, it was classified as diarrhea caused by functional or organic pathologies of the digestive system, such as irritable bowel syndrome, gastrointestinal dysfunction, intestinal flora disorders, and other diseases. With the accelerated pace of modern life, increased work pressure, and poor lifestyle habits, the proportion of people with diarrhea induced by kidney-yang deficiency, and the gradual deterioration of the spleen and stomach were increasing year by year, mainly manifesting as loose diarrhea or prolonged diarrhea with cold extremities. Meanwhile, it was generally accompanied by other diseases that affected the quality of life and imposed a huge economic burden on the state and society.

The modeling method of adenine combined with *Folium sennae* was one of the common diarrhea model.^2^ When the body ingested adenine in high doses, the high concentration of adenine was converted to 2,8-dihydroxyadenine, which was extremely insoluble in water, by the action of xanthine oxidase. This metabolite was deposited in the renal tubules and renal interstitial, forming a foreign body granulomatous inflammation, blocking the lumen of the renal tubules, and causing a cystic dilatation of the corresponding tubular lumen. As the disease progresses, a large number of nephrons were lost, leading to renal failure. Adenine metabolites not only caused renal failure due to mechanical obstruction of renal tubules but also inhibited the activities of various enzymes related to the metabolism of sugar, fat, and protein in renal tissue due to the toxicity of the products, affecting the energy metabolism of renal tissue, resulting in a series of kidney-yang deficiency symptoms such as chills, less movement, decreased food intake, and decreased body weight.^3^ As a bitter-cold laxative commonly used in traditional Chinese medicine (TCM), *Folium sennae* was administered to mice to form the pathological basis of “excessive consumption of cold products damaged spleen-yang,” in order to get closer to the clinical pathogenesis.^4^ Liu et al^5^ pointed out that adenine combined with* Folium sennae* modeling caused mental lethargy, increased urine output, loose feces, weight loss, and reduced food intake in rats with diarrheal irritable bowel syndrome. Although this complex modeling method avoided the limitations of single-factor modeling and improved the success rate of the model by combining different modeling methods, the difference in modeling dose and modeling times also had an obvious impact on the construction of stable disease models during this combined medicine modeling process.^6^

**Figure 1.**
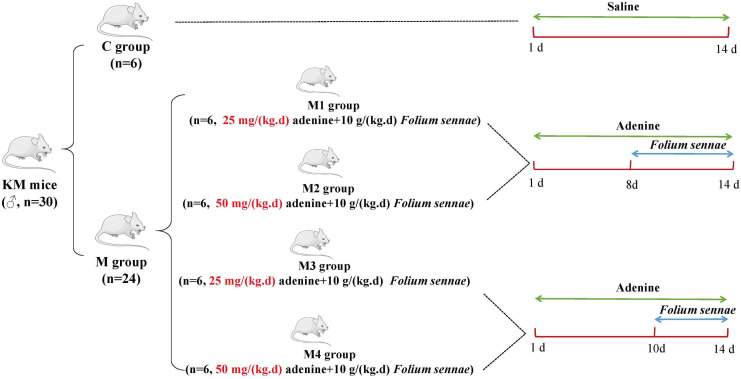
Modeling flow chart of adenine combined with *Folium sennae*.

In this study, adenine combined with *Folium sennae* was used as common modeling factor to construct an animal model of diarrhea. The effects of different modeling doses and modeling times on diarrhea in mice were investigated from the perspectives of macro characterization, organ indexes, blood biochemical indexes, intestinal enzyme activities, and microbial activities, with a view to obtaining a modeling protocol that would better match the current pathogenesis of diarrhea in humans. By comparing the effects of different modeling methods on the kidney function and intestinal biological characteristics of mice with diarrhea, this study laid a foundation for the future development of kidney–intestinal related therapy (Figure 1).

## Materials and Methods

### Animals

Thirty-week-old specific-pathogen-free (SPF) Kunming male mice, weighing 18-22 g, were purchased from Hunan Slaccas Jingda Laboratory Animal Company (Hunan, China) with license number ZS-202105110016. All mice were maintained at room temperature (23-25°C) and relative humidity (50%-70%) with a 12-hour light/dark cycle in the laboratory animal center of Hunan University of Chinese Medicine. The experimental protocols involved animals and their health care approved by the Institutional Animal Care and Use Committee of Hunan University of Chinese Medicine; Xiang. (SYXK (X) 2019-0009) and were performed in strict accordance with the Guide for the Care and Use of Laboratory Animal of National Institute of Health (NIH) (Bethesda, MD, USA).

### Medicines

Adenine (Changsha; Yaer Biology Co., LTD, EZ2811A135); *Folium sennae* (First Hospital of Hunan University of Chinese Medicine, 2005302).

### Adenine Mixture and *Folium sennae* Decoction Preparation

25 mg/(kg/day) adenine suspension: 10.5 mg of adenine in 8.4 mL of sterile water, ready to use. 50 mg/(kg/day) adenine suspension: 18 mg of adenine in 7.2 mL of sterile water, ready to use. To 187.2 g of *Folium sennae*, 300 mL of water was added to soak for 20 minutes, the solution was brought to boil and gently heated for 20 minutes. Then, the drug solution was filtered out with gauze, 200 mL of water was added to the drug residue, and the filtrate was decocted in the same way. Finally, the 2 decoctions were mixed and concentrated to 187.2 mL with a concentration of 1 g/mL and stored at 4°C for future use.

### Animal Grouping and Model Preparation

Thirty male mice were randomly distributed into the control (C), model 1 (M1), model 2 (M2), model 3 (M3), and model 4 (M4) groups, with 6 mice in each group. Mice in the M1 and M3 groups were gavaged 25 mg/(kg/day) adenine suspension and mice in the M2 and M4 groups were gavaged with 50 mg/(kg/day) adenine suspension, 0.4 mL/each, once a day, for 14 days. From the eighth day onwards, M1 and M2 groups received *Folium sennae* decoction (10 g/(kg/day) (0.4 mL/each) once daily for 7 days. M3 and M4 groups were given *Folium sennae* decoction (10 g/(kg/day) (0.4 mL/each) from the 10th day once daily for 5 days.^7,8^ C group was given an equal volume of sterile water administration, once daily for consecutive 14 days.

### Model Evaluation Criteria

According to the clinical manifestations of diarrhea with deficiency of kidney-yang, combined with relevant references,^9^ the diagnostic criteria of macro symptoms in mice diarrhea with deficiency of kidney-yang were as follows: (1) loose feces; (2) decreased appetite and body weight; (3) cold extremities, curved and arched back; (4) a dull and lustrous coat; (5) easy fatigue and lethargy. (1) to (3) were the main symptoms, and (4) and (5) were the concurrent symptoms. The presence of 2 main symptoms and 2 concurrent symptoms could be considered as the basic condition of diarrhea with deficiency of kidney-yang. On the basis of these macro symptoms, the relevant biochemical indexes, including serum creatinine, urea nitrogen, and uric acid levels in mice, were combined to provide a reliable basis for the evaluation of the evidence model.

### Observation of Symptoms and Signs

During the experiment, symptoms and signs of mice were observed and recorded. One day before the end of the experiment, the symptoms and signs of mice in each group were scored according to the basic theory of TCM combined with the symptoms and signs of diarrhea with kidney-yang deficiency,^10^ and the number of mice corresponding to each symptom and sign level in each group was evaluated. Symptoms and signs at all levels similar to those of the C group, grade 1; decreased autonomic activity, lethargy and soft, and well-formed feces, grade 2; slow reaction, depression, body hair sparse, hair (claw, tail) less luster and thin, and soft shape feces, grade 3; cold extremities, curved and arched back, no shiny hair (paws, tail), and feces thin soft not forming, grade 4.

### Body Weight Detection

Body weight of mice was measured and recorded on days 1, 4, 7, 10, and 13 from the beginning of modeling.

### Blood Biochemical Index Detection

After modeling, blood was collected from the eyes of mice under sterile conditions, and the blood samples were sent to the First Hospital of Hunan University of Chinese Medicine for the serum creatinine, urea, and uric acid measurement.

### Thymus and Spleen Index Detection

All mice were sacrificed quickly by cervical dislocation and placed on a super-clean workbench. Under aseptic conditions, the spleen and thymus were taken out and the connective tissues of the thymus and spleen were removed. The surface liquid was dried on clean filter paper. The thymus and spleen of mice were weighed, and the thymus and spleen indexes were calculated. Spleen index = spleen weight (mg)/total live mice weight (g); thymus index = thymus weight (mg)/total live mice weight (g).

### Intestinal Enzyme Activity and Intestinal Microbial Activity Detection

The abdominal cavity of mice was dissected under aseptic conditions and the small intestinal contents were removed. The contents were collected using sterilized forceps. Each group was weighed with 3 g samples of intestinal contents, put into a sterilized centrifugal tube containing glass beads, and the total weight was calculated. After collecting the intestinal contents, the small intestine was cut open along the long axis, the residual intestinal contents were rinsed with saline, and the intestinal wall tissue was blotted with filter paper to remove excess water. The intestinal mucosa was scraped and collected with a sterilized coverslip. Each group was weighed with 3 g samples of the intestinal mucosa, put into a sterilized centrifugal tube containing glass beads, and the total weight was calculated. The crude enzyme solution of intestinal content samples and intestinal mucosal samples were collected respectively. DNS (3,5-dinitrosalicylic acid) colorimetry was used to determine the activities of amylase, sucrase, and xylanase. Lactase activity was determined by the o-Nitrophenyl-β-D-Galactopyranoside (ONPG) method.^11^ Intestinal microbial activity was determined by the fluorescein diacetate (FDA) method.^12^

### Statistical Analysis

The data obtained from each group were expressed as mean ± standard deviation. Statistical analysis was performed by 1-way analysis of variance followed by Tukey’s test using Statistical Package for the Social Sciences v 21.0 (IBM Corp., Armonk, NY, USA). *P* < .05 means there was a significant difference, and *P* < .01 means the difference was very significant.

## Results

### Symptoms and Signs of Diarrhea Mice

During the modeling period, the mental state and autonomous activity of mice in the C group were normal, with smooth hair and a sensitive reaction. The mice in the model groups were in poor mental condition, unresponsive, curved and arched back, cold extremities, sparse and dull hair, damp bedding, dirty perianal area, and loose feces that stuck to the bedding. Mice in the M2 group showed the most obvious. The grading results of symptoms and signs in each group are shown in Table 1.

### Body Weight of Diarrhea Mice

**Figure 2.**
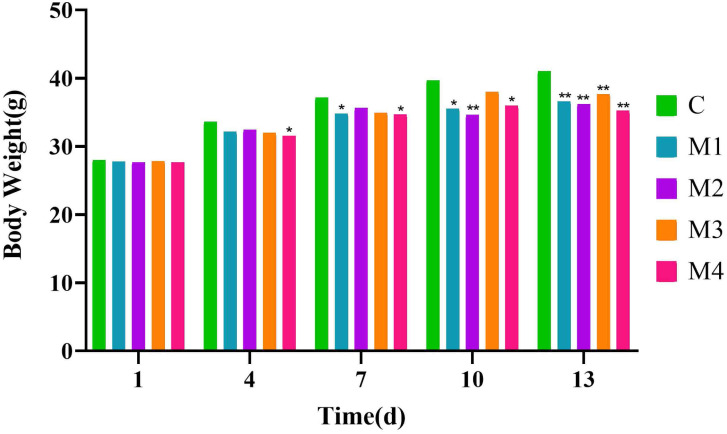
Effects of adenine combined with *Folium sennae* modeling on the body weight of diarrhea mice. Data were expressed as mean ± standard deviation, n = 6, ^*^P < .05, ^**^ P < .01.

With the increase in modeling time, the body weight of mice in all groups was higher than the initial body weight, and the C group had the largest increase (Figure 2). From the fourth day of modeling, the body weight in the model groups decreased compared with that of the C group in the same period, and the decrease in the body weight became more and more obvious with the increase in modeling time. On the 13th day of modeling, mice in the model groups were significantly lower than that in the C group (*P* < .01; *P* < .01; *P* < .01; *P* < .01), the above results indicated that adenine combined with *Folium sennae* inhibited the body weight of mice in the process of modeling.

### Blood Biochemistry Indexes of Diarrhea Mice

**Figure 3.**
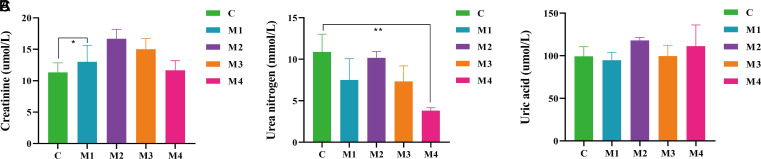
Effects of adenine combined with *Folium sennae* modeling on the blood biochemistry indexes of diarrhea mice. (A) Serum creatinine content. (B) Serum urea nitrogen content. (C) Serum uric acid content. Data were expressed as mean ± standard deviation, n = 6, ^*^P < .05, ^**^P < .01.

When detecting the creatinine content in the serum of mice (Figure 3A), it was found that creatinine levels in the M1, M3, and M4 groups presented an improvement trend in comparison with the C group (*P* < .05; *P >* .05; *P >* .05), and the M2 group increased markedly (*P* < .05). The serum urea nitrogen levels in the M1, M2, and M3 groups all exhibited a trend of reduction compared to the C group (*P >* .05; *P >* .05; *P >* .05), with a distinct reduction in the M4 group (*P* < .01) (Figure 3B). Subsequently, we compared the serum uric acid levels of mice in each group (Figure 3C). Compared with the C group, the levels of uric acid in the M1 group were lower (*P >* .05), while in the M2, M3, and M4 groups, the expression of the uric acid was up-regulated (*P >* .05; *P >* .05; *P >* .05), with the highest increase in the M2 group (*P >* .05). Adenine combined with *Folium sennae* modeling caused damage to the renal function of mice, as evidenced by an increase in the creatinine and a decrease in the urea nitrogen levels. The reason for the different changes in serum uric acid levels due to different modeling methods might be related to the individual differences in mice and uneven drug intake.

### Viscera Indexes of Diarrhea Mice

**Figure 4.**
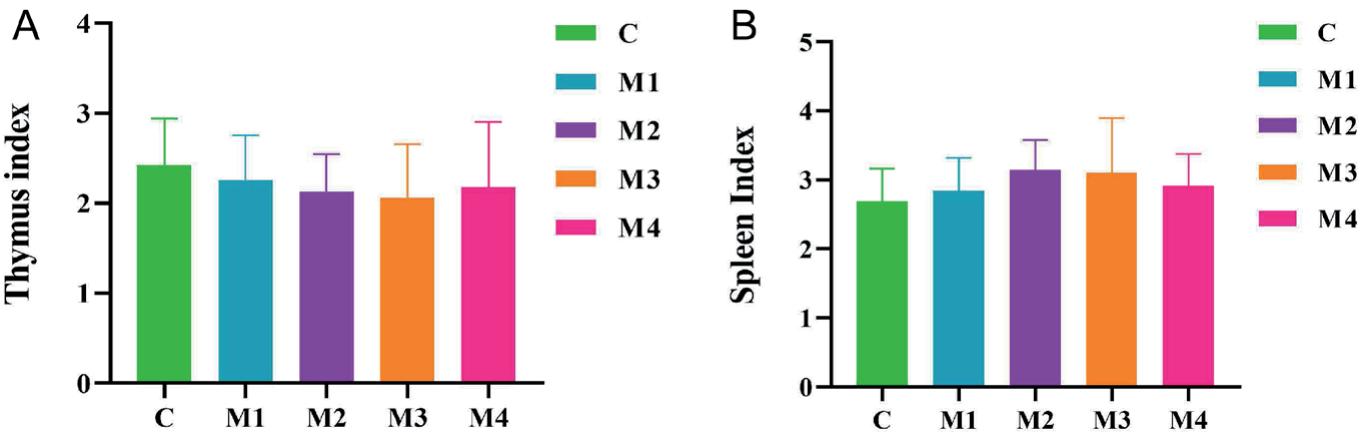
Effects of adenine combined with *Folium sennae* modeling on the viscera indexes of diarrhea mice. (A) Thymus index. (B) Spleen index. Data were expressed as mean ± standard deviation, n = 6.

The thymus index of mice was trending downward after modeling (*P >* .05; *P >* .05; *P >* .05; *P >* .05), with the M2 group showing the greatest decrease (Figure 4A). The spleen index in the model groups had an increasing trend compared with that in the C group (*P >* .05; *P >* .05; *P >* .05; *P >* .05), and the M2 group seemed to be more pronounced (Figure 4B). Briefly, adenine combined with *Folium sennae* modeling reduced thymus index and increased spleen index. Besides, different modeling methods had different effects on the thymus and spleen index in mice.

### Intestinal Enzyme Activities of Diarrhea Mice

**Figure 5.**
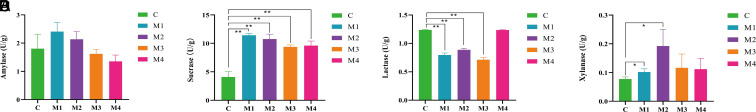
Effects of adenine combined with *Folium sennae* modeling on the intestinal contents enzyme activities of diarrhea mice. (a) Amylase activity. (b) Sucrase activity. (c) Lactase activity. (d) Xylanase activity. Data were expressed as mean ± standard deviation, n = 6, ^*^P < .05, ^**^P < .01.

In our experiments, amylase, sucrase, lactase, and xylanase were measured in the intestinal contents and mucosa of mice. In Figure 5, we could see the effect of adenine combined with *Folium sennae* modeling on the enzyme activities in the intestinal contents of mice. Compared to the C group, the amylase in the intestinal contents was increased in the M1 and M2 groups (*P >* .05; *P >* .05) and decreased in the M3 and M4 groups (*P >* .05; *P >* .05) (Figure 5A). The sucrase content was significantly higher in the model groups (*P* < .01; *P* < .01; *P* < .01; *P* < .01) (Figure 5B). Lactase levels were obviously less in the M1, M2, and M3 groups (*P* < .01; *P* < .01; *P* < .01), with a trend toward lower levels in the M4 group (*P >* .05) (Figure 5C). Xylanase exhibited an increasing trend with some groups showing no statistically significant differences (Figure 5D). The above results suggested that modeling caused some increase in sucrase and xylanase activity and a decrease in lactase in the intestinal contents of mice, which demonstrated different trends in the different modeling methods.

**Figure 6.**
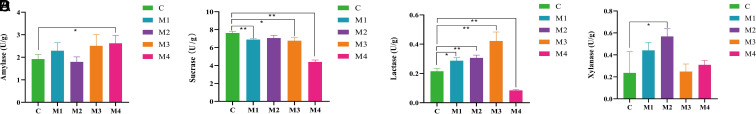
Effects of adenine combined with *Folium sennae* modeling on the intestinal mucosa enzyme activities of diarrhea mice. (A) Amylase activity. (B) Sucrase activity. (C) Lactase activity. (D) Xylanase activity. Data were expressed as mean ± standard deviation, n = 6, ^*^P < .05, ^**^P < .01.

In Figure 6, we could see the effect of adenine combined with *Folium sennae* modeling on the enzyme activities in the intestinal mucosa of mice. The amylase content in the intestinal mucosa was not obviously increased in the M1, M3, and M4 groups in comparison with the C group (*P >* .05; *P >* .05; *P* < .05) and decreased in the M2 group (*P >* .05) (Figure 6A). Sucrase content was reduced in model mice (*P* < .01; *P >* .05; *P* < .05; *P* < .01) (Figure 6B). Notably, the M1, M2, and M3 groups evidenced upregulated levels of lactase (*P* < .05; *P* < .01; *P* < .01), while the M3 group evidenced downregulated levels of lactase (*P* < .01) (Figure 6C). The increase in xylanase, however, was not as pronounced (*P >* .05; *P* < .05; *P >* .05; *P >* .05) (Figure 6D). In a word, modeling caused an increase in xylanase activity and a decrease in sucrase activity in the intestinal mucosa. Amylase and lactase manifested different trends in the different modeling methods.

### Intestinal Microbial Activity of Diarrhea Mice

**Figure 7.**
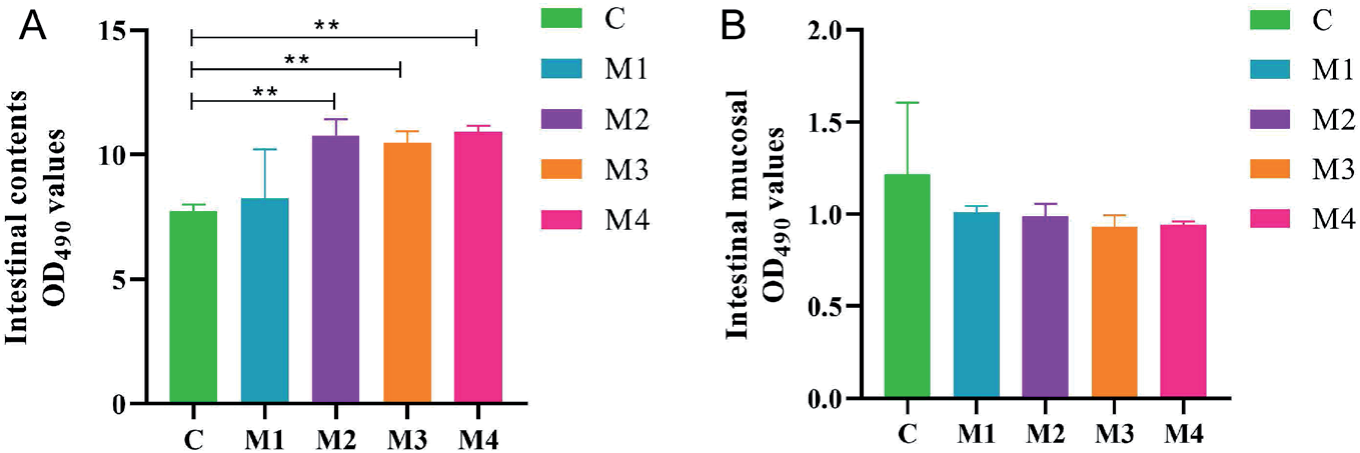
Effects of adenine combined with *Folium sennae* modeling on the intestinal microbial activity of diarrhea mice. (A) Microbial activity of intestinal contents. (B) Microbial activity of intestinal mucosa. Data were expressed as mean ± standard deviation, n = 6, ^*^P < .05, ^**^P < .01.

The microbial activity of intestinal contents of mice after modeling was markedly higher than that of the C group (*P* < .01; *P* < .01; *P* < .01; *P* < .01) (Figure 7A). On the contrary, the intestinal mucosal microbial activity induced the reduction compared with the performance of the control (*P >* .05; *P >* .05; *P >* .05; *P >* .05) (Figure 7B). It could be seen that modeling caused changes in the microbial activity of the intestinal contents and mucosa of mice. The effect of different modeling methods on microbial activity in different regions of the intestine was different, suggesting that adenine combined with *Folium sennae* modeling increased the total microbial load of intestinal contents and decreased the total microbial load of intestinal mucosa in mice.

## Discussion

Most scholars have always emphasized that the establishment of models should pay attention to the animal body surface characteristics as the basis for model evaluation.^13^ During the experiment, it was observed that mice in the model groups showed poor mental condition, lethargic sleep, sparse hair, soft feces, or even curved and arched back, cold extremities, damp bedding, dirty perianal area and loose feces that stuck to the bedding. While mice in the C group demonstrated no corresponding performance, meanwhile, the body weight of mice in the model groups decreased with the increase of the modeling time compared with that of C group during the same period (*P* < .01; *P* < .01; *P* < .01; *P* < .01), suggesting that adenine combined with *Folium sennae* had an inhibitory effect on the body weight of mice and that the model mice were successful in replicating the macroscopic characterization of diarrhea.

With the gradual penetration of modern medical research, laboratory biochemical indexes also began to appear in the auxiliary evaluation indexes of diarrhea with kidney-yang deficiency model. Among them, serum creatinine better reflected the sensitivity of early renal injury. Creatinine rose significantly when the glomerular filtration rate was reduced by more than one-third and was one of the sensitive indicators of impaired renal function.^14^ In our study, the creatinine level in the serum of mice after modeling was higher than that of the C group and the increase in the M2 group was significant (*P* < .05), indicating that the mice had early kidney injury after modeling, and the kidney injury in the M2 group was more serious. The changes in serum urea nitrogen depended mainly on the rate of protein catabolism in the body and the excretory capacity of the kidneys. Urea nitrogen was excreted in the urine by glomerular filtration, of which about 40% was recollected by the renal tubules. When the renal parenchyma was damaged, the glomerular filtration rate decreased and urea nitrogen increased.^15^ Combined with the results of our experiment, the urea nitrogen content of mice did not increase after modeling but decreased, and the M2 group decreased the least. This might be related to the degree of absorption of different doses of adenine in mice and the fact that the target organ of adenine was the kidney and the effect on serum was cumulative damage following renal failure. Simultaneously, mice in the M2, M3, and M4 groups caused an improvement in the uric acid level, of which M2 group was more obvious. On the whole, different modeling methods had different effects on the contents of serum creatinine, urea nitrogen, and uric acid in mice, and the M2 group presented more obvious effects.

Evidence pointed out that diarrhea with kidney-yang deficiency involved the neurological, endocrine, reproductive, and immune systems, mainly manifesting as generalized hypofunction and dysfunction of the neuro–endocrine–immune network, one of the main pathologies being malfunction of the immune system.^16^ The spleen and thymus were vital immune organs in the body. The thymus was capable of producing a large number of thymocytes, which was important for the establishment of the body’s immune function and the recovery of the regulatory function after the loss of immune function. It also secreted a variety of thymic hormones to participate in cellular immune function, enhance anti-tumor, anti-infection ability.^17^ The spleen, as an important site of heterologous immune response in the body, could filter most tumor cells and harmful microorganisms.^18^ The results of this experiment showed that adenine combined with *Folium sennae* decreased the thymic index and increased the splenic index, demonstrating that the modeling process impaired the cellular immunity of mice but caused different effects on humoral immunity, which might be related to individual differences in mice and tolerance to the modeling drugs and the exact reasons needed to be further explored. This decrease in thymus index and increase in spleen index was most pronounced in the M2 group. It also reflected that in the process of modeling with different doses of adenine combined with different days of *Folium sennae* modeling, the effects of modeling dose and days used in the M2 group on the immune function of mice were more prominent.

The small intestine was the main site of food digestion and absorption, and small intestinal disaccharidases (e.g. lactase, sucrase) were widely distributed in the intestinal mucosa and contents. They played a crucial role in carbohydrate utilization by breaking down disaccharides into monosaccharides, which were then transported across the membrane into the blood.^19^ Lack of disaccharide enzyme would make disaccharide digestion, absorption disorder. Lactase and sucrase deficiencies lead to excess carbohydrate accumulation in the small intestine, increasing the intestinal osmotic load, leading to an increase in intestinal luminal fluid and causing osmotic diarrhea. Excess fermentation products enriched intestinal air pressure and distension of the small intestine leading to bloating and abdominal pain.^20^ Lactase and sucrase were important intestinal enzymes associated with diarrhea. Hui et al^21^ preformed that lactase activity in intestinal contents and mucosa of model mice was decreased. Xie et al^22^ denoted that the activity of sucrase in intestinal contents and mucosa of mice with diarrhea model of bacterial imbalance was also decreased.^[Bibr b22-tjg-34-1-4]^ In our study, lactase in intestinal contents and sucrase in the intestinal mucosa of mice decreased after modeling, which was consistent with previous experimental studies. However, sucrase in intestinal contents of mice in the model groups was increased, and lactase in intestinal mucosa in the M1, M2, and M3 groups was increased compared to the C group. The analysis of the reasons for this elevated level was related to the differences in the expression of sucrase and lactase in different parts of the mice intestine as a result of different modeling methods. In addition, it might also be related to the pathways of intestinal sucrase and lactase production. Studies have evinced that intestinal microorganisms synthesized lactase and sucrase in a small amount. In the process of diarrhea, intestinal mucosal damage and bacterial imbalance were common intestinal dysregulation mechanisms.^23^ Some pathogenic or opportunistic bacteria became overgrown due to the lack of antagonism from probiotics, resulting in an imbalance in the intestinal flora. In this imbalance, the diversity of intestinal microorganisms was disturbed and some sucrase- and lactase-producing microorganisms were affected, but the specific influencing factors still needed to be further explored. Amylase was an important digestive enzyme in the intestinal tract and was mainly secreted by the body itself. Hu et al^24^ studied the effect of spleen deficiency diarrhea caused by *Folium sennae* on the amylase activity in the intestinal contents of mice and manifested that the amylase activity of mice with spleen deficiency diarrhea was elevated, which was similar to the elevated amylase in the intestinal contents of mice in the M1 and M2 groups in our experimental results. As well, the amylase content in the intestinal mucosa of mice in the M1, M3, and M4 groups also exhibited an elevated trend, which might be a compensatory elevation or self-protection of the organism in response to the reduced energy supply. Considering that xylanase was mainly secreted by intestinal microorganisms,^25^ the xylanase activity in the intestinal contents and mucosa of the mice in the model groups was increased compared with that in C group, suggesting that the combination of adenine and *Folium sennae* induced a stimulating effect on the microorganisms in the intestinal contents and mucosa of the mice, resulting in a disruption of the original intestinal microbial balance.

Considering that intestinal flora secreted a large number of hydrolytic enzymes to achieve efficient hydrolysis of FDA, which was the main way for intestinal microorganisms to metabolize food and drugs. FDA hydrolytic enzyme activity could, to a certain extent, reflect the metabolic ability of intestinal microorganisms, and therefore the measurement of FDA hydrolytic activity of intestinal samples showed the overall activity of microorganisms in the intestine of animals.^26^ The previous study of the research group demonstrated that the microbial activity in intestinal contents of diarrhea mice was considerably higher than that in the normal group, but the intestinal mucosal microbial activity in the model groups significantly decreased, suggesting that there were differences in the types and numbers of intestinal contents and mucosal microbes in mice.^27^ In our experiment, the microbial activity in the intestinal contents of model mice was higher than that in C group, while the microbial activity in intestinal mucosa was lower than that in C group, which was similar to previous studies. This further presented that the microbial activity of the intestinal contents and mucosa had different trends, so we could know that the effects of adenine combined with *Folium sennae* modeling on different parts of the intestine were different. Of course, modeling increased the metabolic capacity of intestinal contents and reduced the metabolic capacity of intestinal mucosal microorganisms.

In summary, the effect of different modeling doses and the number of days on diarrhea were different. In terms of macro characterization, blood biochemistry, organ indexes, intestinal contents, and mucosal enzymatic activity and microbial activity, the use of 50 mg/kg adenine by gavage for 14 days in combination with 10 mL/kg *Folium sennae* for 7 days induced obvious effect on kidney function injury in diarrhea mice. Furthermore, kidney function injury was accompanied by changing in intestinal functional enzyme activity and microbial activity.

## Figures and Tables

**Table 1.** Classification of Symptoms and Signs in Each Group

Group	Number (n)	Number of Mice Corresponding to Each Symptom and Sign Level (n)			
		Grade 1	Grade 2	Grade 3	Grade 4
C	6	6	0	0	0
M1	6	0	2	4	0
M2	6	0	1	2	3
M3	6	0	3	3	0
M4	6	0	2	2	2
